# A New Basal Sauropodomorph Dinosaur from the Lower Jurassic Navajo Sandstone of Southern Utah

**DOI:** 10.1371/journal.pone.0009789

**Published:** 2010-03-24

**Authors:** Joseph J. W. Sertich, Mark A. Loewen

**Affiliations:** 1 Department of Anatomical Sciences, Stony Brook University, Stony Brook, New York, United States of America; 2 Utah Museum of Natural History, Salt Lake City, Utah, United States of America; Ecole Normale Supérieure de Lyon, France

## Abstract

**Background:**

Basal sauropodomorphs, or ‘prosauropods,’ are a globally widespread paraphyletic assemblage of terrestrial herbivorous dinosaurs from the Late Triassic and Early Jurassic. In contrast to several other landmasses, the North American record of sauropodomorphs during this time interval remains sparse, limited to Early Jurassic occurrences of a single well-known taxon from eastern North America and several fragmentary specimens from western North America.

**Methodology/Principal Findings:**

On the basis of a partial skeleton, we describe here a new basal sauropodomorph dinosaur from the Lower Jurassic Navajo Sandstone of southern Utah, *Seitaad ruessi* gen. et sp. nov. The partially articulated skeleton of *Seitaad* was likely buried post-mortem in the base of a collapsed dune foreset. The new taxon is characterized by a plate-like medial process of the scapula, a prominent proximal expansion of the deltopectoral crest of the humerus, a strongly inclined distal articular surface of the radius, and a proximally and laterally hypertrophied proximal metacarpal I.

**Conclusions/Significance:**

Phylogenetic analysis recovers *Seitaad* as a derived basal sauropodomorph closely related to plateosaurid or massospondylid ‘prosauropods’ and its presence in western North America is not unexpected for a member of this highly cosmopolitan clade. This occurrence represents one of the most complete vertebrate body fossil specimens yet recovered from the Navajo Sandstone and one of the few basal sauropodomorph taxa currently known from North America.

## Introduction

The Early Jurassic (201.5–176 Ma) was an interval of major global climatic and biotic transition that included the diversification of many vertebrate groups. Among these were the sauropodomorphs, a diverse group of herbivorous dinosaurs that would remain among the dominant herbivores in many terrestrial ecosystems through the close of the Mesozoic, a period of more than 140 million years. The basal members of Sauropodomorpha, often referred to as the ‘prosauropods,’ achieved a nearly global distribution by the Early Jurassic, with taxa known from Africa [1], [2], [3], [4], [5], [6], [7], [8], [9], Antarctica [10], Asia [5], [11], [12], [13], [14], [15], India [5]; North America [16], [17], [18], and South America [19], [20].

Basal sauropodomorph remains are scarce in North America. Whereas basal sauropodomorph taxa are well known and globally widespread through the Late Triassic, their remains are conspicuously absent from North American Triassic terrestrial assemblages [21], [22], [23]. The Early Jurassic record of eastern North America includes several specimens of the well known taxon *Anchisaurus* (*Ammosaurus*) from the Lower Jurassic Portland Formation of the Newark Supergroup in the eastern United States [17], [24], [25] and undescribed remains from the Lower Jurassic McCoy Brook Formation in Nova Scotia [18], [26]. By comparison, the Early Jurassic sauropodomorph record of western North America is sparse and fragmentary despite an abundance of exposed terrestrial sedimentary units from this time period. Western North American basal sauropodomorph remains are known from the Lower Jurassic Glen Canyon Group of the Colorado Plateau, including a skull from the base of the Kayenta Formation of Arizona [2], [6], [16], [27] and fragmentary articulated remains from the top of the Navajo Sandstone of Arizona [17], [24], [28], [29], [30], [31]. The recent discovery [32] of a partially articulated specimen from the Navajo Sandstone of southern Utah represents the most complete basal sauropodomorph yet recovered from western North America and one of the few diagnosable specimens from continental North America.

## Methods

### Field Methods and Preparation

The holotype specimen described here was originally discovered and reported to the UMNH by local artist Joe Pachek. The specimen was extracted from the base of a near-vertical cliff wall using a diamond blade on a gas-powered industrial cut-off saw. Trenching around the specimen block required repeated 10 cm deep cuts in a 10 cm grid around the specimen, with removal of blocks of matrix with hammer and chisel. During excavation, the main block naturally split in two along the medial surface of the ventral abdominal wall. The blocks were prepared using pneumatic air scribes and needles under magnification. Due to the delicate preservation of the fossil bone and the articulated nature of the specimen, only the tibia and pelvis were completely removed from the major blocks.

### Imaging

Computed tomography (CT) of the pelvis was undertaken to reconstruct overall morphology using a Siemens Sensation 64 scanner at 120 Kv (3 mm slice interval) at the University of Utah Medical Center.

### Terminology

We employ traditional, or “Romerian,” anatomical and directional terms over veterinary alternatives [33]. For example, “anterior” and “posterior” are used as directional terms in lieu of the veterinary alternatives “cranial” and “caudal.” Identification of vertebral laminae follow recent recommendations [34].

### Institutional Abbreviations

**MCZ**, Museum of Comparative Zoology, Cambridge, Massachusetts, USA; **MNA**, Museum of Northern Arizona, Flagstaff, Arizona, USA; **UMNH**, Utah Museum of Natural History, Salt Lake City, Utah, USA; **UCMP**, University of California Museum of Paleontology, Berkeley, California, USA.

## Results

### Systematic Paleontology

#### Systematic hierarchy

Dinosauria Owen, 1842 [35] *sensu* Padian and May 1993 [36]

Saurischia Seeley, 1887 [37] *sensu* Gauthier 1986 [38]

Sauropodomorpha von Huene, 1932 [39] *sensu* Galton and Upchurch 2004 [5]

### *Seitaad* gen. nov

#### Etymology

*Séít‘áád* (Diné/Navajo), a mythological sand ‘monster’ of Diné folklore that buried its victims in dunes.

#### Type Species

Seitaad ruessi.

### *Seitaad ruessi* sp. nov

urn:lsid:zoobank.org:act:FFA6DE17-5A7F-4D86-A176-51D740BCCA5F

**Figure 1:**
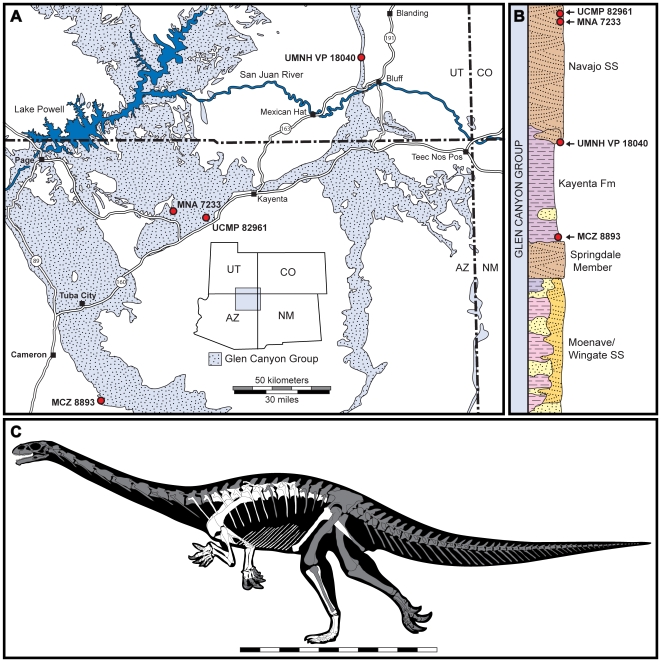
Map and stratigraphic section of the Glen Canyon Group and skeletal reconstruction of *Seitaad ruessi*. Map showing localities of sauropodomorph remains recovered from the Lower Jurassic Glen Canyon Group of southern Utah and northern Arizona (A) including the type locality of *Seitaad ruessi* (UMNH VP 18040). Surface outcrops of the Glen Canyon Group are modified after [77]. Approximate stratigraphic position of all sauropodomorph remains from the Kayenta Formation and Navajo Sandstone (B) including *Seitaad ruessi* (UMNH VP 18040). Stratigraphy of the Glen Canyon Group is modified after [42]. Skeletal reconstruction (C) of *Seitaad* with known elements indicated in white and missing elements indicated in gray. [planned for page width]

Figures 1C–[Fig pone-0009789-g002] [Fig pone-0009789-g003] [Fig pone-0009789-g004] [Fig pone-0009789-g005] [Fig pone-0009789-g006] [Fig pone-0009789-g007] [Fig pone-0009789-g008] [Fig pone-0009789-g009] [Fig pone-0009789-g010] 11

#### Etymology

In honor of the young artist, poet, naturalist, and explorer Everett Ruess (1914–1934?), who mysteriously disappeared in 1934 while exploring southern Utah.

#### Holotype

UMNH VP 18040; articulated partial postcranial skeleton including portions of 11 dorsal vertebrae, 16 dorsal ribs, both pectoral girdles, a nearly complete left and partial right forelimb, partial pelvis, partial left hind limb, and partially articulated array of gastral ribs.

#### Type locality

Comb Ridge, San Juan County, Utah (Fig. 1). The specimen was recovered from UMNH VP Locality 191 at ground level in a slot canyon below remains of the “Eagle's Nest” cliff dwelling.

#### Horizon and Age

UMNH VP 18040 was preserved in a bed of massive sandstone at the base of the Navajo Sandstone, one meter above an interbedded mudstone and sandstone facies of the Kayenta Formation. The Navajo Sandstone is the uppermost unit of the Glen Canyon Group with an age no earlier than Pleinsbachian [31]. The Glen Canyon Group was deposited in a large retroarc basin, with the crossbedded eolian Navajo Sandstone in a complex gradational contact with the underlying fluvially-dominated Kayenta Formation [40], [41], [42].

#### Diagnosis

*Seitaad ruessi* is a non-eusauropod sauropodomorph dinosaur distinguished from other basal saurpodomorphs by the following combination of characters (autapomorphies marked by *): mid-dorsal neural arches subequal to or taller than respective centra; presence of distinct plate-like medial process of the scapula contributing posteriorly to glenoid surface*; prominent deltopectoral crest offset proximally from head of humerus by distinct proximally directed hook*; proximally convex and laterally expanded proximal surface of metacarpal I*; pubis with transversely narrow and elongate apron and relatively small obturator foramen; and enlarged pedal ungual present on digit II.

*Seitaad ruessi* can be further distinguished from the basal sauropodomorphs *Panphagia* and *Saturnalia* on the basis of its elongate and narrow scapular blade and from *Asylosaurus* and *Efraasia* on the basis of its enlarged distal carpal I overlapping distal carpal II, its wide metacarpal I inset into the carpus, and its strongly twisted first phalanx of digit I. *Seitaad* can be distinguished from the plateosaurian taxa *Plateosaurus*, *Massospondylus*, *Riojasaurus*, and *Yunnanosaurus* on the basis of its transversely narrow pubic apron and enlarged ungual of pedal digit II; *Jingshanosaurus* by the absence of a fused carpal block uniting distal carpals II-V; *Lessemsaurus* by its narrow strap-like scapular blade; and *Adeopapposaurus* by its transversely wide metacarpal I. *Seitaad* can be distinguished from the eastern North American sauropodomorph taxon *Ammosaurus* on the basis of its robust metacarpal I, enlarged distal carpal I, relatively short and tall dorsal centra, and large calcaneum.

### Description and comparisons

**Figure 2 pone-0009789-g002:**
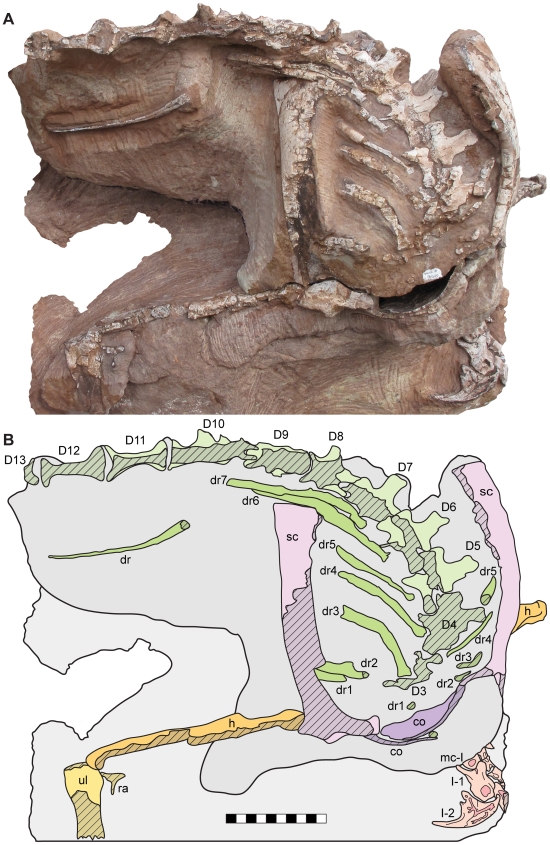
Skeleton of *Seitaad ruessi* (UMNH VP 18040). Shown in anterodorsal view (A) with interpretive line drawing and labels (B). Scale bar equals 10 cm. *Osteological abbreviations*: *co*, coracoid; *D*, dorsal vertebra; *dr*, dorsal rib; *h*, humerus; *ra*, radius; *sc*, scapula; *ul*, ulna; *mc*, metacarpal; *I*, digit one. [planned for page width]

**Figure 3 pone-0009789-g003:**
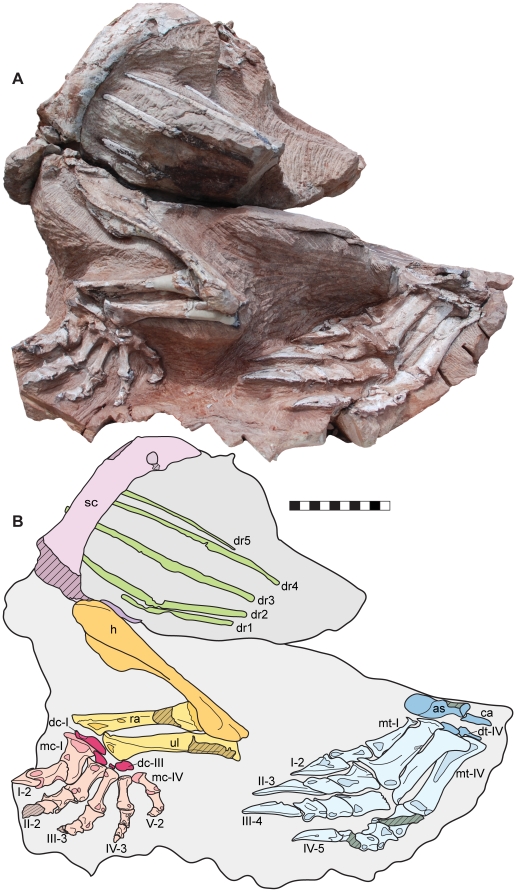
Skeleton of *Seitaad ruessi* (UMNH VP 18040). Shown in left lateral view (A) with interpretive line drawing and labels (B). Scale bar equals 10 cm. *Osteological abbreviations*: *as*, astragalus; *ca*, calcaneum; *dc*, distal carpal; *dr*, dorsal rib; *dt*, distal tarsal; *h*, humerus; *ra*, radius; *sc*, scapula; *ul*, ulna; *mc*, metacarpal; *mt*, metatarsal; *I*, digit one; *II*, digit two; *III*, digit three; *IV*, digit four; *V*, digit five. [planned for page width]

The skeleton of *Seitaad ruessi* (UMNH VP 18040) preserves articulated portions of the axial and appendicular skeleton (Figs. 1C,2,3). Many elements are moderately deformed from post-depositional plastic shear along a roughly posterodorsally/anteroventrally plane.

#### Axial skeleton

The axial skeleton includes portions of eleven articulated dorsal vertebrae (D) and several dorsal ribs preserved in articulation or close association with the vertebrae (Figs. 2,3). Posteriorly, the axial column is laterally flexed to the right. Anteriorly, the column is depressed such that the anterior dorsal vertebrae nearly contact the medial surface of the pectoral girdle. The neural spines and portions of the neural arches of all preserved vertebrae and the proximal portions of most dorsal ribs have been lost to erosion.

**Figure 4 pone-0009789-g004:**
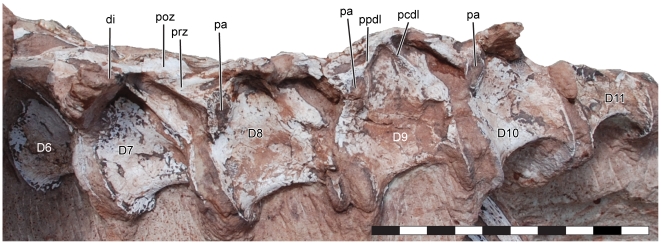
Dorsal vertebrae of *Seitaad ruessi* (UMNH VP 18040). Shown in left lateral view. Scale bar equals 10 cm. *Osteological abbreviations*: *D*, dorsal vertebra; *di*, diapophysis; *pa*, parapophysis; *pcdl*, posterior centrodiapophyseal lamina; *poz*, postzygapophysis; *ppdl*, paradiapophyseal lamina; *prz*, prezygapophysis. [planned for page width]

The vertebrae are interpreted to represent D3 through D13 based on the number and position of preserved ribs and on the proximity of the ventral pelvis. Only morphologically indistinct portions of D3 are preserved on the dorsal surface of the block. Posteriorly, the dorsal surfaces of D4 and D5 are exposed in articulation, though the neural spines and portions of the neural arch have been lost to erosion. The left lateral (Fig. 4) and dorsal surfaces of D6 through D10 are exposed and permit accurate characterization of overall morphology. D11 and D12 are represented only by the ventral portions of the centra, and D13 is represented only by the anteroventral margin of the centrum (Fig. 2).

The dorsal vertebrae are similar in overall morphology to other basal sauropodomorphs. The centra are amphicoelous and relatively short, with a length/height ratio of approximately 3/2, similar to *Adeopapposaurus* [20] and *Massospondylus* [43]. This condition differs from the relatively anterioposteriorly short and dorsoventrally tall dorsal centra of *Riojasaurus* [44] and *Lufengosaurus* [45], and from the relatively elongate and short centra of *Anchisaurus* (*Ammosaurus*) [17], [24]. The anterior and posterior margins of the dorsal centra are strongly flared, resulting in a constricted appearance in lateral view. A broad, shallow fossa is present ventral to the neural arch on the lateral surfaces of the exposed centra.

The neural arches of D8-D10 are dorsoventrally subequal to slightly taller than their respective centra, similar to the conformation in *Adeopapposaurus* and *Unaysaurus* [46], but differing from most other basal sauropodomorphs. Parapophyses are exposed laterally on D8-D10 on the anterior border of the neural arch immediately dorsal to the centrum. The parapophyses are elliptical in outline and dorsoventrally taller than long, with their long axis inclined dorsally and posteriorly. As in other basal sauropodomorphs, a thin laminar paradiapophyseal lamina extends from the parapophysis along the ventral surface of the transverse process. Dorsally, the paradiapophyseal lamina defines the posteroventral margin of a distinct anteroposteriorly elongate anterior chonos on the lateral surface of the prezygapophysis. Ventrally, the paradiapophyseal lamina forms the anterodorsal margin of a distinct triangular anterior infradiapopyseal fossa. The posterodorsal boundary of the anterior infradiapophyseal fossa is demarcated by a thin posterior centrodiapophyseal lamina. The transverse processes are anteroposteriorly centered over the centrum and project laterally. The dorsal surface of the transverse process is continuous with the dorsally flat surface of the prezygapophysis anteriorly and the distinct postzygodiapophyeal lamina posteriorly.

A total of 13 [10 and over use number] dorsal ribs are preserved in articulation or close association with the dorsal vertebrae. The first eight dorsal ribs are preserved on the right side, with ribs articulated with D3 through D7. One rib, likely representing the eighth, is disarticulated and rotated laterally on the right side of the skeleton (Fig. 2). The first five dorsal ribs are preserved on the left side of the skeleton, with ribs three through five articulated with their corresponding vertebrae. In overall morphology, the dorsal ribs are elongate and weakly curved, with an elliptical cross-section. The distal ends of the ribs are not exposed.

#### Appendicular skeleton

The appendicular skeleton includes: nearly the entire left and right pectoral girdles lacking only the anterior margins of the scapulae, coracoids, and the clavicles; portions of both articulated forelimbs, including a nearly complete left forelimb; the ventral pelvis, including both nearly complete pubes; and portions of the left hind limb, including the nearly complete pes.

**Figure 5 pone-0009789-g005:**
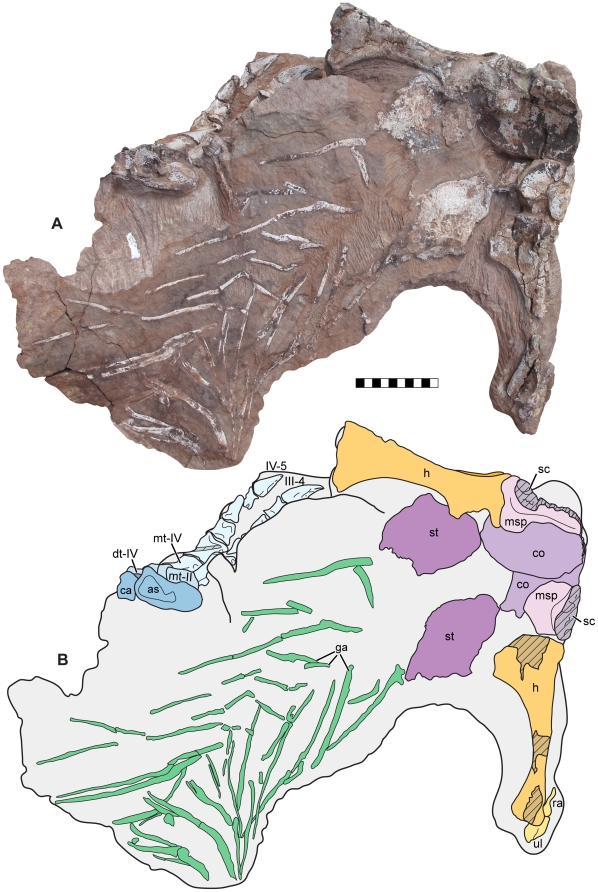
Pectoral and abdominal elements of *Seitaad ruessi* (UMNH VP 18040). Shown in dorsal view (A) with interpretive line drawing and labels (B). Scale bar equals 10 cm. *Osteological abbreviations*: *as*, astragalus; *ca*, calcaneum; *co*, coracoid; *dt*, distal tarsal; *ga*, gastralia; *h*, humerus; *ra*, radius; *sc*, scapula; *ul*, ulna; *msp*, medial scapular process; *mt*, metatarsal; *st*, sternal plate; *III*, digit three; *IV*, digit four. [planned for page width]

**Figure 6 pone-0009789-g006:**
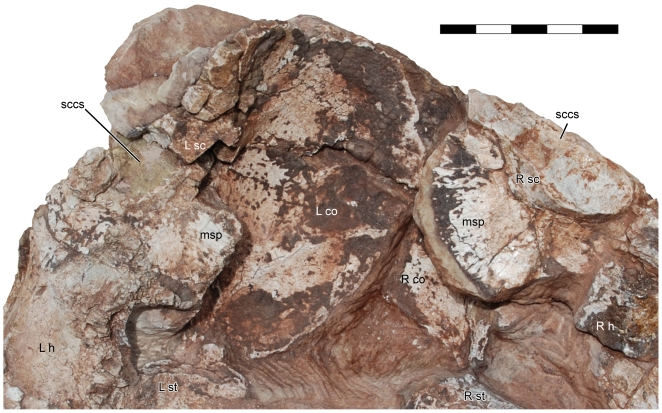
Ventral scapulae and coracoids of *Seitaad ruessi* (UMNH VP 18040). Shown in dorsal view to highlight the plate-like medial scapular processes. Scale bar equals 5 cm. *Osteological abbreviations*: *co*, coracoid; *h*, humerus; *L*, left; *msp*, medial scapular process; *R*, right; *sc*, scapula; *sccs*, cross section of scalupa blade; *st*, sternal plate. [planned for page width]

Both scapulae are well preserved and in articulation with the skeleton (Figs. 2,3,5). The scapula is elongate, mediolaterally flattened, and gently arched laterally, as in other basal sauropodomorphs. The blade is proportionally long and narrow, similar to that of *Adeopapposaurus*, *Plateosaurus* [47], and *Massospondylus*, and differing from the wide scapular blades of *Panphagia* [48], *Lessemsaurus* [49], [50] and *Melanorosaurus* [51]. Dorsally, the blade is weakly expanded anteroposteriorly, producing a gently concave anterior and posterior margin in lateral view. The posterior termination of this expansion forms a sharp corner with the dorsal margin, as in *Plateosaurus* and *Massospondylus* and unlike the more rounded corner of *Adeopapposaurus*. A circular hole, interpreted as pathologic, is present on the dorsal portion of the blade of the left scapula of UMNH VP 18040. Ventrally, the blade expands into the basal portion and its contact with the coracoid (Fig. 5). The anterior margins of the basal portion have been damaged and lost to erosion obscuring the morphology of this region. Anteriorly, the posterior margin of the acromion rises at an angle of less than 65 degrees relative to the long axis of the blade. The basal portion is laterally convex, increasing in thickness mediolaterally toward the coracoid contact. Medially and immediately dorsal to the coracoid contact, a broad semicircular plate of bone projects roughly parallel with the surface of the coracoid (Fig. 6). A similar thickening of this region of the scapula is figured for *Saturnalia* [52], but differs from the plate-like expansion in *Seitaad*. This medial scapular process sweeps posteriorly to contribute, along with the main basal portion of the scapula and the coracoid, to a tri-radiate glenoid surface.

Only the medial surfaces of the strongly overlapping coracoids are visible (Fig. 5). Like the scapula, the anterior margin of the coracoid has been lost to erosion. The medial surface is smooth and concave. As in other sauropodomorphs, the medial margin is strongly convex, curving posteriorly to a sharp posteriorly directed corner. From the posterior corner, the margin turns anteriorly to meet the glenoid surface.

The medial surfaces of both sternal plates are visible, preserved in articulation with the pectoral girdle (Fig. 5). The sternal plates are anteroposteriorly elongate and thin, articulating anteriorly with the posterior corner of the coracoid. Although the sternal plates of other sauropodomorph taxa have been described as elliptical in dorsal or ventral view (e.g., *Adeopapposaurus*, *Massospondylus*, and *Yunnanosaurus* [13]), the sternal plates of *Seitaad* have three distinct margins, as in *Lufengosaurus* [45]. The medial margin is smoothly arched, beginning at its anterior contact with the coracoid and extending posteriorly to a sharp intersection with the lateral margin. The lateral margin can be separated into two distinct sections, a straight irregularly scalloped section and a smooth anterolaterally directed section. A distinct ridge extends posteromedially over the dorsal surface of the sternal plate, originating at the contact between the two portions of the lateral margin, and separating the medial surface into two distinct planes.

**Figure 7 pone-0009789-g007:**
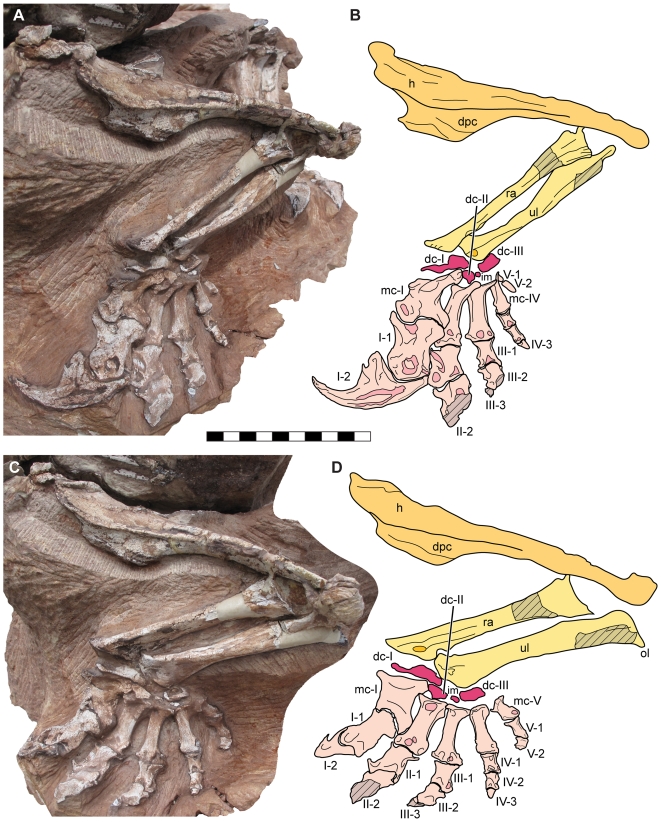
Articulated left arm of *Seitaad ruessi* (UMNH VP 18040). Shown in anterolateral view (A) and lateral view (C) with interpretive line drawing and labels in anterolateral view (B) and lateral view (D). Scale bar equals 10 cm. *Osteological abbreviations*: *dc*, distal carpal; *dpc*, deltopectoral crest; *h*, humerus; *im*, intermedium; *ol*, olecranon process; *ra*, radius; *ul*, ulna; *mc*, metacarpal; *I*, digit one; *II*, digit two; *III*, digit three; *IV*, digit four; *V*, digit five. [planned for page width]

The humerus of *Seitaad* is similar in overall morphology to those of other basal sauropodomorphs. Left and right humeri are preserved in articulation with the pectoral girdle and are exposed along their posterior and lateral surfaces (Figs. 2,3,5,7). The proximal humerus is expanded mediolaterally, with an ellipsoidal proximal head. Medially, the proximal surface is deflected sharply forming a proximomedially directed medial tuberosity. Distal to the medial tuberosity, the humerus is strongly medially concave in posterior view. The deltopectoral crest is prominent, extending anteriorly from the anterolateral edge of the humerus, and extending approximately 50% of the length of the humerus. The anterior edge of the deltopectoral crest is rugose, with a straight proximodistally directed margin. Proximally, the deltopectoral crest is sharply offset from the shaft of the humerus forming a proximally directed spine (Fig. 7A,B). Laterally, a prominent longitudinal ridge extends along the base of the deltopectoral crest to form the lateral margin of the humerus, is similar to the condition in *Unaysaurus* and, to a lesser extent, in *Massospondylus*, *Asylosaurus* [53], and *Riojasaurus*. Distal to the deltopectoral crest, the humeral shaft is constricted and elliptical in cross-section. The distal humerus expands mediolaterally from this constriction, with a large, robust radial condyle.

The radius is a morphologically simple element, with a length 0.67 times that of the humerus. The proximal end is expanded relative to the shaft, with a simple planar articular surface. The shaft is elliptical cross-section, twisting distally approximately 80 degrees relative to the proximal expansion. The distal articular surface is weakly convex and strongly inclined at an angle of approximately 45 degrees relative to the long axis of the shaft. This morphology differs from other basal sauropodomorphs (e.g., *Adeopapposaurus*, *Massospondylus*, *Plateosaurus*, *Saturnalia*, *Unaysaurus*), in which the distal end is offset from the long axis at an angle greater than 70 degrees. However, this may be accentuated by plastic deformation of the distal end in UMNH VP 18040 that is similar to the distortion of this region observed in *Efraasia* [54].

Portions of both ulnae, including the complete left ulna, are preserved (Fig. 7). In general morphology, the ulna is similar to that of other basal sauropodomorphs, with a length 0.69 times the length of the humerus. The proximal end is triangular in outline, with a shallow depression on the anterolateral surface of the proximal shaft for articulation with the proximal radius. This depression is bounded laterally by a short, rounded lateral crest. Posteriorly, the proximal end expands to form a short, rounded olecranon similar in morphology to those of other basal sauropodomorphs except *Saturnalia* in which it is very large. The shaft is elliptical in section, twisting toward the distally expanded distal articular surface. The distal articular surface is smoothly convex.

The carpus preserves three ossified distal carpals and a small ossified intermedium (Fig. 7). Distal carpal I is robust, with a mediolateral width greater than the mediolateral width of the proximal surface of metacarpal I, similar to other basal sauropodomorphs but in contrast to the relatively smaller distal carpal I of *Adeopapposaurus*. The proximal surface is weakly convex and irregular, with a marked proximodistal thickening laterally toward its articulation with the distal ulna. Distally, distal carpal I articulates with metacarpal I and distal carpal II over a weakly convex surface. Distal carpal II is much smaller, only 0.40 times the mediolateral width of distal carpal I. Within the carpus, distal carpal II is closely articulated with the proximolateral expansion of metacarpal I medially, distal carpal I proximally, and the proximal surface of metacarpal II distally. A small, ossified, irregularly shaped intermedium is exposed lateral to distal carpal II, situated proximal to the medial half of the articular surface of metacarpal III. Laterally, the large distal carpal III is centered proximal to metacarpal IV. The proximal surface of distal carpal III is concave for articulation with the distal ulna and the distal surface is flat to weakly convex for articulation with metatarsals III, IV, and V.

The complete articulated left metacarpus and metacarpals I-III of the right metacarpus are preserved (Fig. 7). Metacarpal I is short and robust, with a proximodistal length 0.77 times the length of metacarpal II and a midshaft width nearly twice that of metacarpal II (1.18 proximal width/length ratio). Proximally, metacarpal I is inset into the carpus, with a prominent proximolateral process articulating laterally with distal carpal II and the proximal surface of metacarpal II. Though an inset metacarpal I is present in many basal sauropodomorphs (e.g., *Adeopapposaurus*, *Massospondylus*, *Plateosaurus*), the proximolateral process of *Seitaad* is expanded proximally and laterally beyond the lateral extent of the lateral distal condyle and possesses a distinctly convex proximal surface for articulation with distal carpal I. Distally, metacarpal I is asymmetric, with a distally expanded lateral condyle similar to that of many basal sauropodomorphs. Metacarpal II is the longest of the metacarpals, with moderate expansions of the proximal and distal articular surfaces. Metacarpals III, IV, and V decrease in size sequentially retaining the same overall proportions, with metacarpal V 0.76 times the length of metacarpal IV. Proximally, all five metacarpals articulate tightly along their medial and lateral surfaces. Distally, the metacarpals diverge, with metacarpal V set nearly perpendicular to metacarpal II as preserved (Fig. 7).

The complete manual phalangeal formula of *Seitaad* is unknown, with the distal portions of digits II and III missing (2-?-?-3-2). The first phalanx of digit I is greatly enlarged and asymmetric; the transverse axis of the distal articular surface twisted laterally approximately 60 degrees relative to the mediolateral axis of the proximal end. This morphology differs from the significantly less twisted first phalanges of *Efraasia* and *Asylosaurus* but is generally similar to the morphology present in *Massospondylus*, *Unaysaurus*, *Plateosaurus*, *Yunnanosaurus*, and *Anchisaurus*. The second phalanx of digit I consists of a robust, laterally compressed and strongly curved ungual oriented medially within the manus by the combined asymmetry of metacarpal I and of the first phalanx. A deep longitudinal ridge runs along the medial surface of the ungual distally from the proximal articular facet. Proximally, a well developed flexor tubercle is present on the ventral edge. Portions of the first two phalanges are preserved in digits II and III. The widths of the proximal ends are subequal in size relative to the mediolaterally expanded distal ends. Deep collateral pits are present on the lateral surfaces of the distal condyles. A small medial fragment of the third phalanx of digit III is also present, though more distal phalanges and unguals have been lost in digits II and III. Digit IV preserves three complete phalanges. The first two are similar in overall morphology to the proximal phalanges of digits II and III, though with shallow collateral pits and deeply divided distal condyles. The distalmost ossified phalanx is small and subquadrangular in dorsal outline. Digit V preserves two complete phalanges. The first phalanx is elongate, 0.90 times the length of metacarpal V, and strongly constricted between the proximal and distal surfaces. The distal phalanx is elongate and paddle-shaped, with a flat proximal articulation and a rounded dorsoventrally compressed body. Morphologically, the distal phalanx is much longer than the proximodistally short distal phalanges of other basal sauropodomorphs including *Efraasia*, *Massospondylus*, and *Plateosaurus*.

**Figure 8 pone-0009789-g008:**
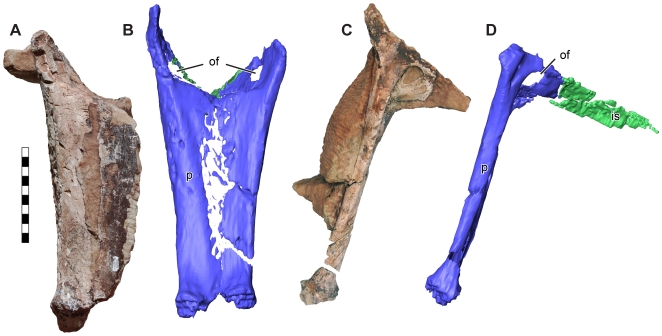
Partial pelvis of *Seitaad ruessi* (UMNH VP 18040). Shown in anterior/dorsal view (A) and left lateral view (C) with CT-reconstructions in anterior/dorsal view (B) and left lateral views (D). Scale bar equals 10 cm. *Osteological abbreviations*: *of*, obturator foramen; *p*, pubis; *is*, ischium. [planned for page width]

Much of the dorsal pelvic girdle, including the entire ilium and most of the ischium, has been lost to erosion so that only the pubes are well preserved (Fig. 8). The distal pubic blade is elongate and anteroposteriorly compressed, contacting the contralateral blade along its entire length to form a pubic apron (Fig. 8B). Laterally, each distal blade is thickened, thinning to a lamina toward its medial contact and thus forming a teardrop-shaped transverse section. The pubic apron of *Seitaad* narrows distally, differing from the transversely broad pubic aprons of many basal sauropodomorphs (e.g., *Panphagia*, *Saturnalia*, *Efraasia*, *Massospondylus*, *Melanorosaurus*, *Plateosaurus*, *Riojasaurus*) yet similar to the narrow aprons of *Adeopapposaurus* [20] and *Anchisaurus* (*Ammosaurus*) [17], [24]. The distal end of the blade is expanded anteroposteriorly to form a small rugose ‘boot.’ Proximally, the pubis is thickened toward its contact with the ilium, with a subtriangular cross section. Medially, the proximal end twists to form a dorsoventrally compressed ventral lamina that contacts its antimere medially and the ischium posteriorly. A small elliptical laterally directed obturator foramen (Fig. 8C,D) is present on the lateral surface of the pubis, similar in proportion to the obturator of *Adeopapposaurus* [20]. Only the ventral laminae of the ischia are preserved Both laminae meet at the midline and contact the ventral laminae of the pubes along an anteriorly convex suture.

**Figure 9 pone-0009789-g009:**
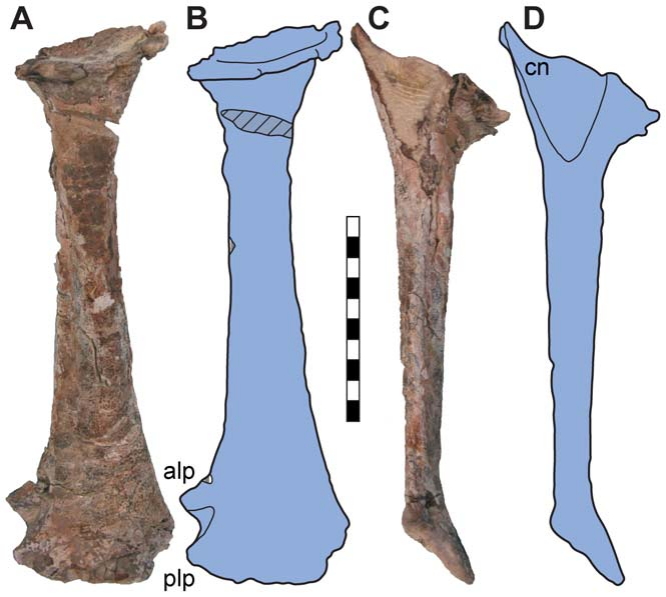
Left tibia of *Seitaad ruessi* (UMNH VP 18040). Shown in anterior view (A) and lateral view (C) with interpretive line drawing and labels in anterior view (B) and lateral view (D). Scale bar equals 10 cm. *Osteological abbreviations*: *alp*, anterior lateral process; *cn*, cnemial crest; *plp*, posterior lateral process. [planned for column width]

The complete left tibia is moderately distorted by plastic deformation directed along a proximal/anterior to distal/posterior axis. Generally, the tibia is morphologically similar to the tibiae of other basal sauropodomorphs (Fig. 9). A well developed cnemial crest continuous with the medial surface is present, divided by a shallow fossa from the external prominence laterally. The shaft is straight and elliptical in section, compressed anteroposteriorly. The distal end is expanded mediolaterally, with a well developed anterolateral process and more distally expanded posterolateral process.

**Figure 10 pone-0009789-g010:**
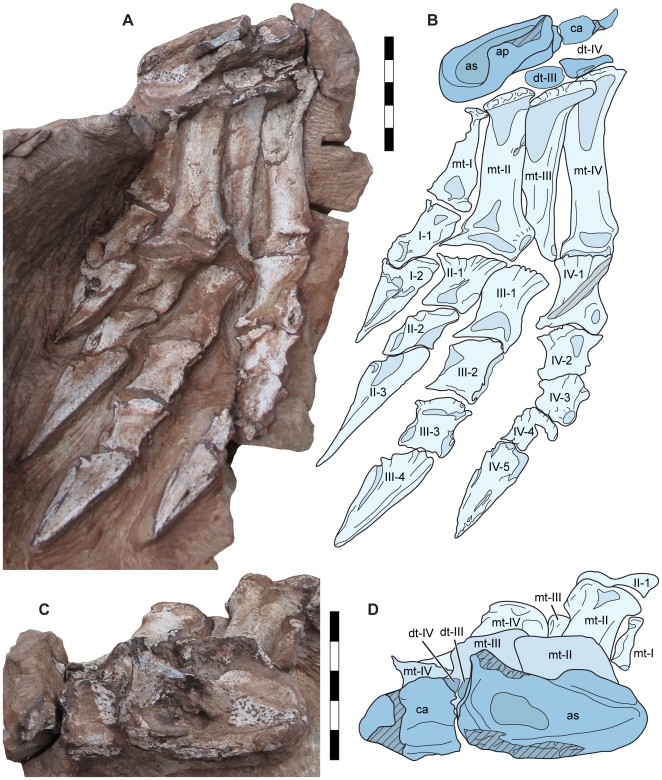
Left pes of *Seitaad ruessi* (UMNH VP 18040). Pes shown in dorsal view (A) with interpretive line drawing and labels (B). Tarsus shown in proximal view (C) with interpretive line drawing and labels (D). Scale bar equals 5 cm. *Osteological abbreviations*: *ap*, ascending process; *as*, astragalus; *ca*, calcaneum; *dt*, distal tarsal; *mt*, metatarsal; *I*, digit one; *II*, digit two; *III*, digit three; *IV*, digit four; *V*, digit five. [planned for page width]

The astragalus is subtrapezoidal in dorsal view, with a rounded medial margin expanding laterally to a straight lateral contact with the calcaneum (Fig. 10B), with a similar morphology to *Plateosaurus*, *Massospondylus*, and *Adeopapposaurus* but differing from the mediolaterally short astragalus of *Panphagia* and *Saturnalia* and the anteroposteriorly narrow astragalus of *Efraasia*. The proximal surface consists of an open elliptical fossa with a laterally sloping floor. A subtriangular ascending process originating along the anterolateral margin forms the anterior wall of the proximal fossa; anteromedially, the proximal fossa is open along an anteriorly sloping surface. The lateral surface of the astragalus is weakly concave for contact with the distal fibula.

The calcaneum consists of a proximodistally flattened subtriangular plate approximately 0.56 times the mediolateral width of the astragalus. The proximal surface is weakly concave for contact with the distal fibula. Distally, the calcaneum articulates with distal tarsal IV.

Two distal tarsals are preserved in the articulated tarsus (Fig. 10). Distal tarsal III is centered over the medial surface of metatarsal III and distal tarsal IV is proximal to the lateral edge of metatarsal III and most of the proximal surface of metatarsal IV. Both distal tarsals articulate proximally with the astragalus and calcaneum.

The first four metatarsals are preserved in articulation (Fig 10A,B). The proximal surfaces are distorted and obscured proximally by the astragalus and distal tarsals. Metatarsal I is the shortest, 0.62 times the length of metatarsal II. Metatarsal II is slightly shorter than the subequal third and fourth metarsals, approximately 0.94 times the length of metatarsal III. Proximally, the metatarsals are mediolaterally expanded, with a laterally imbricating pattern of articulation. The shafts are robust and moderately compressed anteroposteriorly, with elliptical sections. Distally the metatarsals expand to a form a mediolaterally broad distal ginglymus with deep collateral pits on both medial and lateral surfaces. The distal ginglymus of both metatarsals I and II is directed medially at an angle of 10 to 15 degrees relative to the proximal ends. Metatarsal V is either not preserved or not exposed.

The pedal phalanges of the first four digits of the left pes are completely preserved in articulation with a phalangeal formula of 2-3-4-5-? (Fig. 10). The first phalanx of digit I is asymmetric, the transverse axis of its distal end twisted relative to its proximal end. The other pedal phalanges of digits II, III, and IV are similar in morphology, with proximally expanded proximal articular surfaces, and expanded distal condyles with shallow collateral pits. Unlike other basal sauropodomorphs, in which the ungual of digit I is the largest (e.g., *Adeopapposaurus*, *Massospondylus*, *Lufengosaurus*), the ungual of digit II is 1.4 times the length of ungual I. The unguals are moderately laterally compressed and triangular, with longitudinal grooves running along the medial and lateral margins.

#### Dermal ossifications

An intact, though distorted gastral array is visible on the medial surface of the ventral abdominal wall (Fig. 5). Distinction between lateral and medial elements is not evident, though an imbricated pattern of overlap between medial elements [55] is evident. The gastralia are elongate and gently curved, with an elliptical, dorsoventrally compressed cross section. Anteriorly, the gastralia are in close association with the sternal plates.

## Discussion

### Preservation

**Figure 11:**
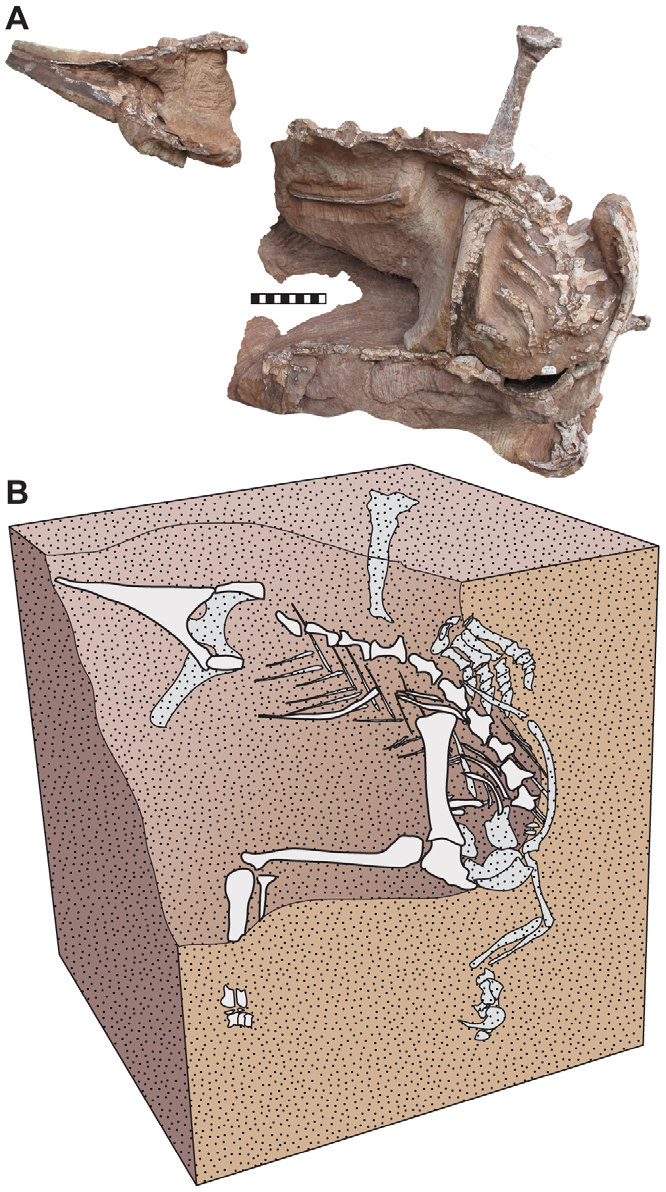
Position of the remains of *Seitaad ruessi* (UMNH VP 18040) . Position reconstructed as discovered relative to bedding (A) with reconstruction of the specimen (B). White indicates bones exposed on the surface. Scale bar equals 10 cm. [planned for column width]

Many vertebrate body fossils from the Navajo Sandstone consist of articulated to partially articulated skeletons deposited in interdune or dune facies [31]. The holotype skeleton of *Seitaad* (UMNH VP 18040) represents one of the most complete vertebrate fossils yet recovered from the Navajo Sandstone and its unique preservation warrants brief discussion here. As exposed on the surface of the canyon wall upon discovery, the remains of *Seitaad* include the cross sections of eroded bone from dorsal vertebrae 3 through 13, as well as the proximal pubes and ventral ischia (Fig. 11). The right forelimb was exposed as an impression along with an accompanying thin film of cortical bone from the scapula through metacarpals I-III. Some distal phalanges of the left manus were destroyed during excavation. The only preserved element that is not in near articulation is the left tibia, which is rotated 90 degrees on its long axis and displaced by 10 cm from articulation with the astragalus. There is no sign of a left fibula or digit V. Anterior and posterior portions of the skeleton—including the skull, cervical series, caudal region, dorsal pelvis, and femur—are presumed to have been eroded away prior to discovery.

UMNH VP 18040 is preserved in a one meter thick massive sand bed at the base of a 200 m cliff of Navajo Sandstone. The homogenous sand bed represents the first long term onset of Navajo Sandstone in the area. This contact between the Kayenta Formation and Navajo Sandstone illustrates the local switch from a fluvial environment, periodically inundated with tongues of sand erg deposits, to a permanent onset of long term dune activity [56], [57]. Locally, the lower Navajo Sandstone is characterized by cross-bedded sandstone interpreted as eolian dune sets. Slumped and massive sandstone beds, sedimentary packages that are locally common in the lower 10 meters of the Navajo Sandstone, are associated with these dune sets and likely indicate dune slumps or collapsed dune faces. These deposits have been identified in other parts of the unit regionally [56]. The sedimentary package preserving UMNH VP 18040 is petrologically identical to laterally equivalent sandstones, but lacks both small and large scale indicators of eolian deposition including foreset cross-bedding. The sand bed that preserves UMNH VP 18040 is laterally equivalent to eolian dune sets and is interpreted as a local dune collapse deposit.

Taphonomic preservation of the specimen, with articulation of every preserved bone in place, suggests that the specimen was held together by soft tissues at the time of burial. The orientation of the specimen (40 degrees from horizontal on the mediolateral plane and 85 degrees on the sagittal plane, in a head down position), together with the high degree of articulation, is further suggestive of dune collapse preservation. The partial disassociation of the left tibia and the absence of the fibula and pedal digit V suggest that the animal was dead prior to burial.

### Navajo Sandstone sauropodomorphs

Other fragmentary sauropodomorph remains from the Navajo Sandstone have been referred to *Anchisaurus* (*Ammosaurus*) [17], [30], though these referrals have recently come into question [24]. The first sauropodomorph remains reported from this unit [28], [29], MNA G2 7233 (now cataloged as MNA V743 through MNA V752), consist of portions of several articulated caudal vertebrae, poorly preserved pubes, a partial right tibia, both articulated pedes, and several articulated gastralia. This specimen is clearly not referable to *Seitaad* because it possesses a broad pubic apron and likely represents an indeterminate taxon given its fragmentary preservation [24]. Another partial sauropodomorph from the Navajo Sandstone, UCMP 82961, consists of two cervical vertebrae and cervical ribs, an articulated left manus, fragments of the right manus, several pedal phalanges, and fragments of the pectoral girdle [31]. Previous authors have mistakenly identified a pedal phalanx and ungual as part of digit I of the manus [i.e.], [17], [30,31]. Though incomplete, the manus of UCMP 82961 does compare favorably with *Seitaad*, including a proximally expanded lateral process of metacarpal I and an enlarged distal carpal I overlapping distal carpal II. Unfortunately, the specimen is too incomplete to make a confident diagnosis or referral. However, it does preserve enough features to indicate it belongs to the clade Plateosauria *sensu* Yates [24], which includes *Plateosaurus* and all more derived sauropodomorphs.

### Phylogenetic position

*Seitaad* is considered a distinct taxon based on several morphologic features of the pectoral girdle and forelimb not present in other basal sauropodomorphs. The distinct, plate-like medial process of the scapula contributing posteriorly to the glenoid surface is autapomorphic. A medial thickening of the scapular glenoid region is figured for *Saturnalia* [52], but does not approach the morphology of the semicircular plate present bilaterally in *Seitaad*. Additionally, the prominent offset proximal hook of the deltopectoral crest is autapomorphic for *Seitaad*. In many basal sauropodomorph taxa, the proximal margin of the deltopectoral crest is offset sharply from the proximal articular surface; however, all other taxa lack the accompanying proximal expansion present in *Seitaad*. Finally, the proximally convex and laterally expanded proximal surface of metacarpal I represents a feature that is absent in other basal sauropodomorph taxa. In other basal sauropodomorphs, the proximal end of metacarpal I is laterally inset into the carpus. However, in these taxa, the lateral margin of the proximal surface does not extend beyond the lateral boundary of the lateral distal condyle or project proximally to the degree present in *Seitaad*.

Relationships among basal sauropodomorphs, or ‘prosauropods’ have been historically unstable and controversial with some phylogenetic analyses supporting paraphyly of the group with respect to Sauropoda [e.g.], [38], [46], [49], [50], [58], [59], [60], [61], [62], [63], [64] and other analyses supporting monophyly of all or a reduced subset of ‘prosauropods’ [e.g.], [5], [24], [65], [66], [67], [68], [69], [70,71]. However, recent iterations of both datasets have largely converged in their results, agreeing that ‘prosauropods’ likely represent a paraphyletic assemblage of single lineages and small sub-clades along the stem of Sauropoda [72] though a consensus topology has not yet been achieved. A detailed revision of basal sauropodomorph phylogeny is beyond the scope of this study. In order to assess the phylogenetic position of *Seitaad*, preliminary phylogenetic analyses were performed using two of the most exhaustive datasets published to date representing each of the proposed prosauropod topologies [64], [71], as modified by a recent analysis [72]. Both analyses were performed using TNT v. 1.1 [73] with a heuristic search of 1000 replicates of Wagner trees (using random addition sequences) followed by Tree Bisection and Reconnection (TBR) branch swapping (holding 10 trees per replicate).

**Figure 12:**
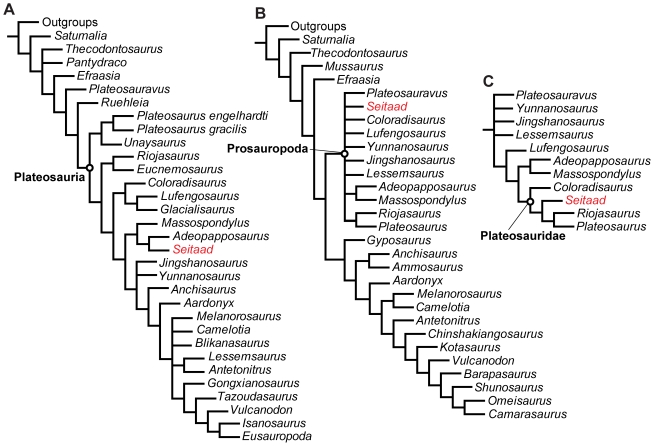
Phylogenetic analyses of basal sauropodomorph dinosaurs including *Seitaad ruessi*. Analyses are based on the datasets of Yates [64] showing the strict consensus of 16 MPTs (A) and Upchurch et al. [71], showing the strict consensus of 18 MPTs (B) and the topology of Prosauropoda in the strict consensus of 8 MPTs with the exclusion of Character 189 (C). Both analyses are modified according to Yates [72] and include additional taxa. Relationships among all non-sauropodomorph taxa (here collapsed into a collective ‘outgroups’) are identical to those recovered in their respective analyses. [planned for page width]

The phylogenetic analysis of Yates [64] represents one of the most exhaustive published to date in support of prosauropod paraphyly with respect to Sauropoda. In addition to the modifications of Yates et al. [72], additional characters and scorings described by Smith and Pol [10] and the South American saupodomorph *Adeopapposaurus* [20] were included in the analysis (Dataset S1). The analysis resulted in the recovery of 16 MPTs, each with a length of 1173 steps, with a consistency index of 0.358 and a retention index of 0.689. The strict consensus of the 16 MPTs is presented in Fig. 12A. *Seitaad* is recovered as sister to *Adeopapposaurus* from the Lower Jurassic Cañón del Colorado Formation of Argentina. This relationship is supported by four unambiguous synapomorphies: height of neural arches subequal to or greater than the centrum height (Character 164: 0→1); minimum scapula width less than 20 percent its length (Character 200: 1→0); length of deltopectoral crest extends between 30 and 50 percent the length of the humerus (Character 207: 2→1); width of conjoined pubes less than 75 percent their length (Character 259: 1→0). The primary difference between this analyses and previous iterations of the dataset [64], [72] is the separation of Massospondylidae (*sensu* [64]) into two separate stem based clades: (*Coloradisaurus* + *Lufengosaurus* + *Glacialisaurus*) and (*Massospondylus* + *Adeopapposaurus* + *Seitaad*).

The analysis of Upchurch et al. [71] represents one of the most comprehensive and complete datasets with results in support of prosauropod monophyly, though the recent modifications to this analysis by Yates et al. [72] recovered a paraphyletic topology with respect to Sauropoda. The analysis was expanded with the addition of the taxa and character modifications of Yates et al. [72] and the inclusion of *Seitaad* and the basal sauropodomorph *Adeopapposaurus* [20] (Dataset S2). The analysis followed the procedures outlined by Upchurch et al. [71] including the treatment of all characters as binary (except character 144 which was treated as an ordered multistate character) and constraining the topology of the non-sauropodmorph outgroup taxa to conform to that of Langer [74]. The phylogenetic analysis resulted in the recovery of 54 most parsimonious trees (MPTs) of 768 steps, with a consistency index of 0.387 and a retention index of 0.659. Exclusion of *Blikanasaurus* from the analysis following Upchurch et al. [71] resulted in the recovery of 18 MPTs of 767 steps, with a consistency index of 0.387 and a retention index of 0.659. Strict consensus of this analysis (Fig. 12B) recovered a *Seitaad* as part of a poorly resolved prosauropod clade in contrast to the recent results of Yates et al. [72]. Support for this clade consists of a single synapomorphy: ratio of proximal width of metacarpal I to length greater than 1.0 (Character 189: 0→1; convergently present in *Melanorosaurus*). With the exception of *Adeopapposaurus*, character 189 is redundant with character 188 in the dataset. Exclusion of character 189 from the analysis reduces the number of MPTs to 8 (762 steps, CI 0.388, RI 0.660) and resolves many of the ingroup relationships among prosauropods in the strict consensus (Fig. 12C), placing *Seitaad* among plateosaurids (*sensu* Upchurch et al. [71]) and sister to a clade that includes *Plateosaurus* and *Riojasaurus*. Support for this clade consists of four unambiguous synapomorphies: strap-shaped scapular blade with subparallel margins in lateral view (Character 157: 1→0; convergently present in *Efraasia*, *Saturnalia*, *Lufengosaurus*, *Shunosaurus*, *Omeisaurus*); caudal margin of acromion arises at less than 65 degrees form the long axis of the scapular blade (Character 158: 1→0; convergently present in *Thecodontosaurus*, *Melanorosaurus*, *Jingshanosaurus*, *Kotasaurus*, *Efraasia*, *Antetonitrus*); craniocaudal length of distal pubic expansion divided by pubis length less than 0.15 (Character 228: 1→0; convergently present in *Saturnalia*, *Efraasia*, *Anchisaurus*, *Antetonitrus*); and ungual on pedal digit I shorter than some pedal phalanges (Character 285: 1→0; convergently present in *Thecodontosaurus* (as *Asylosaurus* [53]), *Saturnalia*, *Massospondylus*). The recovery of a monophyletic Prosauropoda in this modified Upchurch et al. [71] dataset is notable in context of the recent results of Yates et al. [72] that recovered a paraphyletic assemblage of single lineages and small sub-clades along the stem of Sauropoda. *A posteriori* exclusion of *Adeopapposaurus* suggests that the inclusion of this relatively complete taxon in this analysis causes this shift back to a monophyletic assemblage of prosauropods. However, in this analysis, Prosauropoda does consist of a smaller subset of taxa than in the original iterations of the main dataset [71], with *Anchisaurus* (*Ammosaurus*) and *Gyposaurus* found to be closer to Sauropoda in agreement with the topologies of Yates [24], [64] and Yates et al. [72].

The conflicting placement of *Seitaad* among sauropodmorphs in both analyses likely stems from the variable taxon and character sampling among the two analyses and the incomplete nature of the holotype of *Seitaad*. Both analyses are heavily weighted with cranial and vertebral characters that cannot be evaluated for *Seitaad*. Nevertheless, both analyses support inclusion of *Seitaad* in the clade Plateosauria *sensu* Yates [64] as a derived taxon closely related to either *Plateosaurus* and other plateosaruids or *Adeopapposaurus* and *Massospondylus* in a reduced Massospondylidae. Further, the analyses do not support close association between *Seitaad* and the eastern North American taxon *Anchisaurus* (*Ammosaurus*) as has been suggested for other sauropodomorph remains from the Navajo Sandstone [17], [30].

### Biogeographic implications

The recovery of *Seitaad* from the Navajo Sandstone of Utah represents the first diagnosable sauropodomorph material from the Early Jurassic of western North America. Unfortunately, the lack of phylogenetic resolution in analyses of sauropodomorphs including *Seitaad* precludes meaningful biogeographic interpretations. Globally, Early Jurassic basal sauropodomorphs are widely distributed and include taxa from South America (*Adeopapposaurus* [20]), Asia (*Lufengosaurus*, *Jingshanosaurus*. “*Gyposaurus sinensis*”, *Yimenosaurus*, and *Yunnanosaurus* [11], [12], [13], [14], [15], [45]), Antarctica (*Glacialisaurus* [10]), Africa (*Massospondylus*, *Gryponyx africanus*, *Aardonyx* [3], [4], [43], [72]), and eastern North America (*Anchisaurus* [17], [24]). These occurrences can be loosely grouped into ‘eastern’ (Asia) and ‘southern’ (southern South America, southern Africa, Antarctica) regions, with Early Jurassic continental reconstructions placing North America and Europe in an intermediate position between these regions [75]. Previous attempts to draw biogeographic inferences among continental vertebrates from this time period recovered a pattern of worldwide faunal homogeneity [76]. Morphological similarities between *Seitaad* and plateosaurid and massospondylid taxa are therefore not unexpected given the cosmopolitan distribution of other members of the clade and the intermediate position of North America. The possible close relationship between *Seitaad* and the Early Jurassic ‘southern’ taxa *Adeopapposaurus* and *Massospondylus* is notable given previous inferred links between these two areas based on sauropodomorph specimens [2], [6], [16] and theropod specimens [76] from the underlying Kayenta Formation. Ultimately, further resolution of the phylogenetic position of *Seitaad* and a more thorough understanding of other basal sauropodomorph remains from the Kayenta Formation [2], [6], [16], [27] will permit better insight into the role of this biogeographically significant region in analyses of Early Jurassic continental biotas.

### Nomenclatural Acts

The electronic version of this document does not represent a published work according to the International Code of Zoological Nomenclature (ICZN), and hence the nomenclatural acts contained herein are not available under that Code from the electronic edition. A separate edition of this document was produced by a method that assures numerous identical and durable copies, and those copies were simultaneously obtainable (from the publication date listed on page 1 of this article) for the purpose of providing a public and permanent scientific record, in accordance with Article 8.1 of the Code. The separate print-only edition is available on request from PLoS by sending a request to PLoS ONE, 185 Berry Street, Suite 3100, San Francisco, CA 94107, USA along with a check for $10 (to cover printing and postage) payable to “Public Library of Science”.

The online version of the article is archived and available from the following digital repositories: PubMedCentral (www.pubmedcentral. nih.gov/), and LOCKSS (http://www.lockss.org/lockss/). In addition, this published work and the nomenclatural acts it contains have been registered in ZooBank (http://www.zoobank. org/), the proposed online registration system for the ICZN. The ZooBank LSIDs (Life Science Identifiers) can be resolved and the associated information viewed through any standard web browser by appending the LSID to the prefix “http://zoobank.org/”. The LSID for this publication reads as follows: pub:498FF4CB-D04B-409D-B255-AFB09268BDE8

## Supporting Information

Dataset S1 Nexus file of the character-taxon matrix used for the phylogenetic analysis based on Yates [2007] as modified by Yates et al. [2010] and with the additional characters of Smith and Pol [2007].
(0.02 MB TXT)

Dataset S2 Nexus file of the character-taxon matrix used for the phylogenetic analysis based on Upchurch et al. [2007] as modified by Yates et al. [2010].
(0.01 MB TXT)

## References

[1] Cooper MR (1984). A reassessment of *Vulcanodon karibaensis* Raath (Dinosauria: Saurischia) and the origin of the Sauropoda.. Palaeontologia Africana.

[2] Gow CE (1990). Morphology and growth of the *Massospondylus* braincase (Dinosauria: Prosauropoda).. Palaeontologia Africana.

[3] Gauffre FX (1993). The most recent Melanorosauridae (Saurischia, Prosauropoda), Lower Jurassic of Lesotho with remarks on the prosauropod phylogeny.. Neues Jahrbuch für Geologie und Paläontologie Monatshefte.

[4] Barrett PM (2004). Sauropodomorph dinosaur diversity in the upper Elliot Formation (*Massospondylus* range zone: Lower Jurassic) of South Africa.. South African Journal of Science.

[5] Galton PM, Upchurch P, Weishampel DB, Dodson P, Osmólska H (2004). Prosauropoda.. The Dinosauria, second edition.

[6] Sues HD, Reisz RR, Hinic S, Raath MA (2004). On the skull of *Massospondylus carinatus* Owen, 1854 (Dinosauria: Sauropodomorpha) from the Elliot and Clarens formations (Lower Jurassic) of South Africa.. Annals of Carnegie Museum.

[7] Vasconcelos CC, Yates AM (2004). Sauropodomorph biodiversity of the upper Elliot Formation (Lower Jurassic) of southern Africa..

[8] Barrett PM, Yates AM (2006). New information on the palate and lower jaw of *Massospondylus* (Dinosauria: Sauropodomorpha).. Palaeontologia Africana.

[9] Yates AM, Bonnan MF, Neveling J, Chinsamy A, Blackbeard MG (2009). A new transitional sauropodomorph dinosaur from the Early Jurassic of South Africa and the evolution of sauropod feeding and quadrupedalism.. Proceedings of the Royal Society of London B.

[10] Smith ND, Pol D (2007). Anatomy of a basal sauropodomorph dinosaur from the Early Jurassic Hanson Formation of Antarctica.. Acta Palaeontologia Polonica.

[11] Young CC (1941). Acomplete osteology of *Lufengosaurus huenei* Young (gen. et sp. nov.).. Palaeontologica Sinica, series C.

[12] Young CC (1941). *Gyposaurus sinensis* (sp. nov.), a new Prosauropoda from the Upper Triassic Beds at Lufeng, Yunnan.. Bulletin of the Geological Society of China.

[13] Young CC (1942). *Yunnanosaurus huangi* (gen. et sp. nov.), a new Prosauropoda from the Red Beds at Lufeng, Yunnan.. Bulletin of the Geological Society of China.

[14] Bai Z, Yang J, Wang G (1990). *Yimenosaurus*, a new genus of Prosauropoda from Yimen County, Yunnan province.. Yuxiwenebo (Yuxi Culture and Scholarship).

[15] Zhang Y, Yang Z (1994). A Complete Osteology of Prosauropoda in Lufeng Basin Yunnan China.. Jingshanosaurus.

[16] Attridge J, Crompton AW, Jenkins FA (1985). The southern African Liassic prosauropod *Massospondylus* discovered in North America.. Journal of Vertebrate Paleontology.

[17] Galton PM (1976). Prosauropod dinosaurs (Reptilia: Saurischia) of North America.. Postilla.

[18] Fedak TJ (2006). A rich bone bed of sauropodomorph dinosaurs in the Early Jurassic (Hettangian) McCoy Brook Formation.. Journal of Vertebrate Paleontology.

[19] Martinez RN (1999). The first South American record of Massospondylus (Dinosauria: Sauropodomorpha).. Journal of Vertebrate Paleontology.

[20] Martinez RN (2009). *Adeopapposaurus mognai*, gen. et sp. nov. (Dinosauria: Sauropodomorpha), with comments on adaptations of basal Sauropodomorpha.. Journal of Vertebrate Paleontology.

[21] Nesbitt SJ, Irmis RB, Parker WG (2007). A critical re-evaluation of the Late Triassic dinosaur taxa of North America.. Journal of Systematic Palaeontology.

[22] Irmis RB, Nesbitt SJ, Padian K, Smith ND, Turner AH (2007). A Late Triassic Dinosauromorph Assemblage from New Mexico and the Rise of Dinosaurs.. Science.

[23] Nesbitt SJ, Smith ND, Irmis RB, Turner AH, Downs A (2009). A complete skeleton of a Late Triassic saurischian and the early evolution of dinosaurs.. Science.

[24] Yates AM (2004). *Anchisaurus polyzelus* (Hitchcock): The smallest known sauropod dinosaur and the evolution of gigantism among sauropodomorph dinosaurs.. Postilla.

[25] Fedak T, Galton PM (2007). New information on the braincase and skull of *Anchisaurus polyzelus* (Lower Jurassic, Connecticut, USA; Saurischia: Sauropodomorpha): implications for sauropodomorph systematics. In: Barrett PM, Batten DJ, eds. Evolution and Palaeobiology of Early Sauropodomorph Dinosaurs.. Special Papers in Palaeontology.

[26] Shubin NH, Olsen PE, Sues HD, Fraser NC, Sues HD (1994). Early Jurassic small tetrapods from the McCoy Brook Formation of Nova Scotia, Canada.. In the Shadow of the Dinosaurs.

[27] Tykoski RS (2005). Vertebrate paleontology in the Arizona Jurassic.. Mesa Southwest Museum Bulletin.

[28] Brady LF (1935). Preliminary note on the occurrence of a primitive theropod in the Navajo.. American Journal of Science.

[29] Brady LF (1936). A note concerning the fragmentary remains of a small theropod recovered from the Navajo Sandstone in northern Arizona.. American Journal of Science.

[30] Galton PM (1971). The prosauropod dinosaur *Ammosaurus*, the crocodile *Protosuchus*, and their bearing on the age of the Navajo Sandstone of Northeastern Arizona.. Journal of Paleontology.

[31] Irmis RB (2005). A review of the vertebrate fauna of the lower Jurassic Navajo Sandstone in Arizona. In: McCord RD, ed. Vertebrate Paleontology of Arizona.. Mesa Southwest Museum Bulletin.

[32] Loewen MA, Sertich JJ, Sampson SD, Getty MA (2005). Unusual preservation of a new sauropodomorph from the Navajo Sandstone of Utah.. Journal of Vertebrate Paleontology.

[33] Wilson JA (2006). Anatomical nomenclature of fossil vertebrates: standardized terms or ‘lingua franca’?. Journal of Vertebrate Paleontology.

[34] Wilson JA (1999). A nomenclature for vertebral laminae in sauropods and other saurischian dinosaurs.. Journal of Vertebrate Paleontology.

[35] Owen R (1842). Report on British fossil reptiles.Part II.. Report of the British Association for the Advancement of Science 1841.

[36] Padian K, May CL, Lucas SG, Morales M (1993). The earliest dinosaurs..

[37] Seeley HG (1887). On the classification of the fossil animals commonly named Dinosauria.. Proceedings of the Royal Society of London.

[38] Gauthier J, Padian K (1986). Saurischian monophyly and the origin of birds..

[39] von Huene F (1932). Die forrile Reptil−Ordnung Saurischia, ihre Entwicklung und Geschichte.. Monographien zur Geologie und Palaeontologie,Series 1.

[40] Middleton LT, Blakey RC, Brookfield ME, Ahlbrandt TS (1983). Processes and controls on the intertonguing of the Kayenta and Navajo Formations, northern Arizona: eolian–fluvial interactions.. Eolian Sediments and Processes.

[41] Blakey RC, Caputo MV, Peterson JA, Fanczyk KJ (1994). Paleogeographic and tectonic controls on some Lower and Middle Jurassic erg deposits, Colorado Plateau.. Mesozoic Systems of the Rocky Mountain Region, USA.

[42] Lucas SG, Heckert AB, Tanner LH (2005). Arizona's Jurassic vertebrates and the age of the Glen Canyon Group.. New Mexico Museum of Natural History and Science Bulletin.

[43] Cooper MR (1981). The prosauropod dinosaur *Massospondylus carinatus* Owen from Zimbabwe: Its biology, mode of life and phylogenetic significance.. Occasional Papers of the National Museums and Monuments of Rhodesia (Series B).

[44] Bonaparte JF (1972). Los tetrápodos del sector superior de la Formación Los Colorados, La Rioja, Argentina (Triásico Superior).. Opera Lillona.

[45] Young CC (1947). On *Lufengosaurus magnus* Young (sp. nov.) and additional finds of *Lufengosaurus huenei* Young.. Palaeontologica Sinica, series C.

[46] Leal LA, Azevedo SAK, Kellner AWA, Da Rosa ÁAS (2004). A new early dinosaur (Sauropodomorpha) from the Caturrita Formation (Late Triassic), Paraná Basin, Brazil.. Zootaxa.

[47] von Huene F (1926). Vollständige osteologie eines Plateosauriden aus demschwäbischen Keuper.. Geologische und Palaeontologische Abhandlungen Neue Folge.

[48] Martinez RN, Alcober OA (2009). A basal sauropodomorph (Dinosauria: Saurischia) from the Ischigualasto Formation (Triassic, Carnian) and the early evolution of Sauropodomorpha.. Plos One 4.

[49] Bonaparte JF (1999). Evolución de las vértebras presacras en Sauropodomorpha.. Ameghiniana.

[50] Pol D, Powell JE (2005). New information on *Lessemsaurus sauropoides* (Dinosauria, Sauropodomorpha) from the Late Triassic of Argentina.. Journal of Vertebrate Paleontology.

[51] van Heerden J, Galton PM (1997). The affinities of *Melanorosaurus*, a Late Triassic prosauropod dinosaur from South Africa.. Neues Jahrbuch für Geologie und Paläontologie, Monatshefte.

[52] Langer MC, Franca MAG, Gabriel S, Barrett PM, Batten DJ (2007). The pectoral girdle and forelimb anatomy of the stem-sauropodomorph *Saturnalia tupiniquim* (Upper Triassic, Brazil)..

[53] Galton PM (2007). Notes on the remains of archosaurian reptiles, mostly basal sauropodomorph dinosaurs, from the 1834 fissure fill (Rhaetian, Upper Triassic) at Clifton in Bristol, southwest England.. Revue de Paléobiologie, Genève.

[54] Galton PM (1973). On the anatomy and relationships of *Efraasia diagnostic*a (Huene) n. gen., a prosauropod dinosaur from the Upper Triassic of Germany.. Paläontologische Zeitschrift.

[55] Claessens LPAM (2004). Archosaurian respiration and the pelvic girdle aspiration breathing of crocodyliforms.. Proceedings of the Royal Society of London B.

[56] Blakey RC, Peterson F, Kocurek G (1988). Synthesis of late Paleozoic and Mesozoic aeolian deposits of the Western Interior of the United States.. Sedimentary Geology.

[57] Loope DB, Rowe CM (2003). Long-lived pluvial episodes during deposition of the Navajo Sandstone.. Journal of Geology.

[58] von Huene F (1929). Kurze Übersicht über die Saurischia und ihre natürlichen Zusammenhänge.. Paläontologische Zeitschrift.

[59] Colbert EH (1964). Relationships of saurischian dinosaurs.. American Museum Novitiates,.

[60] Romer AS (1968). Notes and comments on vertebrate paleontology..

[61] Bonaparte JF (1969). Comments on early saurischians.. Zoological Journal of the Linnean Society of London.

[62] Bonaparte JF, Padian K (1986). The early radiation and phylogenetic relationships of the Jurassic sauropod dinosaurs, based on vertebral anatomy.. The beginning of the age of dinosaurs.

[63] Yates AM (2003). A new species of the primitive dinosaur *Thecodontosaurus* (Saurischia: Sauropodomorpha) and its implications for the systematics of early dinosaurs.. Journal of Systematic Palaeontology.

[64] Yates AM, Barrett PM, Batten DJ (2007). The first complete skull of the Triassic dinosaur *Melanorosaurus* Haughton (Sauropodomorpha: Anchisauria)..

[65] Cruickshank AR (1975). The origin of sauropod dinosaurs.. South African Journal of Science.

[66] Sereno PC (1989). Prosauropod monophyly and basal sauropodomorph phylogeny.. Journal of Vertebrate Paleontology.

[67] Sereno PC (1999). The evolution of dinosaurs.. Science.

[68] Wilson JA, Sereno PC (1998). Early evolution and higher-level phylogeny of sauropod dinosaurs.. Society of Vertebrate Paleontology Memoir.

[69] Yates AM, Kitching JW (2003). The earliest known sauropod dinosaur and the first steps towards sauropod locomotion.. Proceedings of the Royal Society of London B.

[70] Barrett PM, Upchurch P, Wang XL (2005). Cranial osteology of *Lufengosaurus huenei* Young (Dinosauria: Prosauropoda) from the Lower Jurassic of Yunnan, People's Republic of China.. Journal of Vertebrate Paleontology.

[71] Upchurch P, Barrett PM, Galton PM (2007). A phylogenetic analysis of basal sauropodomorph relationships: implications for the origin of sauropod dinosaurs. In: Barrett PM, Batten DJ, editors. Evolution and Palaeobiology of Early Sauropodomorph Dinosaurs.. Special Papers in Palaeontology.

[72] Yates AM, Bonnan MF, Neveling J, Chinsamy A, Blackbeard MG (2010). A new transitional sauropodomorph dinosaur from the Early Jurassic of South Africa and the evolution of sauropod feeding and quadrupedalism.. Proceedings of the Royal Society of London B.

[73] Goloboff P, Farrish J, Nixon K (2003). http://www.zmuc.dk/public/phylogeny.

[74] Langer MC, Weishampel DB, Dodson P, Osmolska H (2004). Basal Saurischia.. The Dinosauria.

[75] Smith AG, Smith DG, Funnell BM (1994). Atlas of Mesozoic and Cenozoic coastlines..

[76] Smith ND, Makovicky PJ, Pol D, Hammer WR, Currie PJ (2007). The dinosaurs of the Early Jurassic Hanson Formation of the central Transantarctic Mountains: phylogenetic review and synthesis.. U.S. Geological Survey and the National Academies, Short Research Paper.

[77] USGS (2009).

